# A Novel Flow Cytometric Hemozoin Detection Assay for Real-Time Sensitivity Testing of *Plasmodium falciparum*

**DOI:** 10.1371/journal.pone.0061606

**Published:** 2013-04-24

**Authors:** Maria Rebelo, Claudia Sousa, Howard M. Shapiro, Maria M. Mota, Martin P. Grobusch, Thomas Hänscheid

**Affiliations:** 1 Instituto de Medicina Molecular, Faculdade de Medicina de Lisboa, Lisbon, Portugal; 2 Centre de Recherches Médicales de Lambaréné - CERMEL, Albert Schweitzer Hospital, Lambaréné, Gabon; 3 The Center for Microbial Cytometry, West Newton, Massachusetts, United States of America; 4 Department of Infectious Diseases, Centre for Tropical and Travel Medicine, Amsterdam Medical Centre, Amsterdam, The Netherlands; 5 Institute of Tropical Medicine, University of Tübingen, Tübingen, Germany; Kenya Medical Research Institute - Wellcome Trust Research Programme, Kenya

## Abstract

Resistance of *Plasmodium falciparum* to almost all antimalarial drugs, including the first-line treatment with artemisinins, has been described, representing an obvious threat to malaria control. *In vitro* antimalarial sensitivity testing is crucial to detect and monitor drug resistance. Current assays have been successfully used to detect drug effects on parasites. However, they have some limitations, such as the use of radioactive or expensive reagents or long incubation times. Here we describe a novel assay to detect antimalarial drug effects, based on flow cytometric detection of hemozoin (Hz), which is rapid and does not require any additional reagents. Hz is an optimal parasite maturation indicator since its amount increases as the parasite matures. Due to its physical property of birefringence, Hz depolarizes light, hence it can be detected using optical methods such as flow cytometry. A common flow cytometer was adapted to detect light depolarization caused by Hz. Synchronized *in vitro* cultures of *P. falciparum* were incubated for 48 hours with several antimalarial drugs. Analysis of depolarizing events, corresponding to parasitized red blood cells containing Hz, allowed the detection of parasite maturation. Moreover, chloroquine resistance and the inhibitory effect of all antimalarial drugs tested, except for pyrimethamine, could be determined as early as 18 to 24 hours of incubation. At 24 hours incubation, 50% inhibitory concentrations (IC50) were comparable to previously reported values. These results indicate that the reagent-free, real-time Hz detection assay could become a novel assay for the detection of drug effects on *Plasmodium falciparum.*

## Introduction

Resistance of *Plasmodium falciparum* to almost all antimalarial drugs has been observed [1]. In fact, resistance to commonly effective and useful drugs such as chloroquine or sulfadoxine/pyrimethamine has severely compromised their use for malaria control [2]. Alarmingly, resistance to the currently used first-line treatment compounds, the artemisinins, characterized by a prolonged parasite clearance time [3], has already been reported from South-East-Asia. Consequently, detection and monitoring of drug resistance is of paramount importance.

Traditionally, therapeutic efficacy trials are the gold standard for assessing parasite response to antimalarial drugs. The obvious complexity of these trials led to the development of *in vitro* assays [4]. The major *in vitro* phenotypic assays include the WHO schizont maturation microtest [5], the isotope ([3H]-hypoxanthine) incorporation assay [6], the detection of the parasite antigens pLDH [7] or HRP2 [8] by ELISA, and assays using fluorescent DNA dyes, such as SYBR green I [9], YOYO [10], PicoGreen [11] and DAPI [12] with either spectrophotometric or cytometric readout (Table S1).

The development of novel antimalarial compounds hinges on assays to determine the inhibitory effects of drugs on the parasite [13]. Although all these assays have been successfully applied to detect drug effects on the parasite, they all have relevant limitations. For example, the WHO microtest is based on the tedious and subjective microscopic observation of parasite maturation [14]. The [3H]-hypoxanthine assay requires expensive equipment as well as complex isotope handling precautions and radioactive waste management [6]. All these assays require reagents for parasite detection that are often rather expensive and frequently require a cold chain. Importantly, they also need incubation times of 48 up to 96 hours to reliably detect drug effects [15].

Molecular methods are highly desirable, because they do not depend on viable parasites and have the capacity to provide rapid results. Their major drawback is the limited number of known and validated resistance markers [4]. It is important to note that there is currently no specific *in vitro* test to identify artemisinin resistance, as stated by an expert panel in the WHO Global Plan for Artemisinin Resistance Containment (GPARC) [16].

In this scenario, alternative assays that may overcome the limitations previously mentioned are highly desirable. An assay that would not only allow real-time determination of drug effects during a single parasite cycle but could also detect drug effects in a second or even third cycle would certainly be a useful tool, permitting the assessment of inhibitory effects of drugs with different times of action.

Malaria pigment, i.e., hemozoin (Hz), is produced in increasing amounts by the parasite during the erythrocytic cycle and, therefore, constitutes an ideal maturation indicator. Hz, the end product of plasmodial hemoglobin metabolism, has been identified as an important modulator of the host's immune response to *Plasmodium* spp., as a marker for disease severity and prognostic factor for disease outcome, and also as an adjuvant diagnostic tool, of particular use regarding the non-immune traveler [17]–[19]. Hz depolarizes light and can be easily detected thereby without reagents by optical methods including dark-field microscopy [20], polarization microscopy [21] and flow cytometry [22].

In 1999, a study reported that the flow cytometry based full-blood-count analyser, Cell-Dyn® (Abbott, Santa Clara, CA), could detect Hz within leucocytes [23]. More importantly, studies reported that the Cell-Dyn® seemed to detect Hz inside parasitized red blood cells (RBC) [24], [25]. Based on the flow cytometric detection of depolarized side scatter [26], as used in the Cell-Dyn®, we showed that Hz could be detected inside parasitized RBC in *P. berghei* infected rodents [27]. Moreover, *in vitro* parasite maturation, as well as the inhibitory effect of chloroquine and quinine, could be detected after only 6 hours of incubation [27]. Later, we showed that maturation of *P. falciparum* in culture could also be determined [28].

Our present data show how flow cytometric detection of Hz can be used as a novel, reagent-free, real time assay to assess antimalarial drug effects on *P. falciparum*.

## Methods

All reagents were obtained from Sigma Aldrich (St Louis, Mo, USA), unless stated otherwise.

### Flow cytometer modification (depolarized side scatter detection)

The Cyflow® Blue (Partec, Münster, Germany) is a portable (Figure S1), five parameter flow cytometer with blue laser (488 nm) excitation, and detectors for forward scatter (FSC), side scatter (SSC), green fluorescence (FL1), orange fluorescence (FL2) and red fluorescence (FL3). For this study the set-up was modified as described elsewhere [27]. Briefly, two SSC detectors were created, with a 50%/50% beam splitter between them. Then a polarization filter was placed orthogonally (horizontal) to the polarization plane of the laser light (vertical), in front of one of the SSC detectors, allowing the detection of depolarized side scatter (Figure S1). The Cyflow® is equipped with an absolute cell count method (http://www.partec.com/instrumentation/flow-cytometry.html, accessed 3/8/2012), which allows determination of the number of particles in 200 µl of sample. Absolute counts were performed for all experiments to control for possible red blood cell lysis.

### Microscopy

Parasitemia, parasite maturation and the synchronicity of parasites in culture were assessed by light microscopic examination of Giemsa-stained blood smears. Air-dried blood smears were fixed in absolute methanol and stained with Giemsa (Merck, Darsmstadt, Germany) in a 1∶10 dilution in PBS 1×, for 20 minutes.

### *Plasmodium falciparum* continuous cultures

The *Plasmodium falciparum* resistant (Dd2) and susceptible (3D7) strains were grown in recently collected donor erythrocytes in RPMI based complete malaria culture medium (CMCM) according to the recommendations of the Malaria Research and Reference Reagent Resource Center (MR4) [29]. Cultures were maintained at 5% hematocrit, at 37°C in an atmosphere of 5% CO_2_. As uninfected controls, erythrocytes from healthy donors were cultured as described above.

### Synchronizing *Plasmodium falciparum* continuous cultures

Continuous cultures of *P. falciparum* were cultivated until they reached a parasitemia of >2% with a minimum of 50% rings. They were synchronized by adding 5% sorbitol for 10 minutes at room temperature as described elsewhere [30]. Briefly, the culture medium was washed away by centrifuging the culture at 1800 rpm, for 5 minutes. Next, 10 mL of 5% sorbitol was added to the pelleted red blood cells and incubated for 10 minutes, at room temperature. Cultures were washed twice in PBS 1× by centrifugation at 1800 rpm, for 5 minutes. Finally, CMCM was added to the pelleted cells, and the synchronized culture was incubated for another 48 hours, at 37°C in a 5% CO_2_ atmosphere.

### Hemozoin detection sensitivity assay

Ring-stage synchronized cultures (at least 90% of ring forms) at 2.5% hematocrit and at approximately 1% parasitemia were incubated with antimalarial drugs or with CMCM (used for the drug free and uninfected controls) in 24 or 96 well-plates, for 48 hours, at 37°C in a 5% CO_2_ atmosphere.

Quinine, chloroquine, mefloquine, artemisinin, artesunate, and pyrimethamine were purchased from Sigma Aldrich (St Louis, Mo, USA). NITD246 was kindly provided by Dr. Bryan Yeung from the Novartis Institute for Tropical Diseases, Singapore. Stock solutions of chloroquine and quinine were prepared in distilled water, artemisinin and mefloquine in pure methanol (Merck, Darmstadt, Germany), pyrimethamine in absolute ethanol (Merck, Darmstadt, Germany), artesunate in 70% ethanol and NITD246 in pure DMSO.

Doubling concentrations ranging from 6 to 200 nM for chloroquine, 10 to 160 nM for mefloquine, 12 to 200 nM for pyrimethamine and 4 to 64 nM for artesunate and artemisinin were tested. For NITD246 concentrations of 0.1, 0.2, 1 and 2 nM were used, while for quinine concentrations of 3, 12, 50, 200 and 800 nM were tested.

For each flow cytometric measurement approximately 100,000 events were analyzed. A volume of 5 µL of the blood suspension present in the wells was stained with SYBR green 1×, as described below. To determine the best time point for IC50 calculation, measurements were done at 6 hour intervals over 48 hours for the majority of the drugs tested, except for pyrimethamine which was measured again at 72 hours. All samples were analyzed in triplicate and, for each drug, at least three different experiments were performed. In order to investigate possible inoculation effects, artesunate and artemisinin were also investigated at a lower parasitemia of 0.4 and 0.7%, respectively.

To assess if renewing artesunate would influence its effect on parasite growth, cultures were washed and fresh artesunate, at a concentration of 8 nM, was added every 12 hours, during 48 hours of incubation.

To investigate the detection limit of the novel Hz assay, ring-stage synchronized cultures with parasitemias of 0.05%, 0.1%, 0.3%, 0.5%, 0.6% and 1% were incubated for 48 hours.

Finally, to assess the performance of the Hz assay with low parasitemias, ring-stage synchronized cultures at 0.3% parasitemia were incubated for 72 hours with increasing concentrations of chloroquine, artesunate and pyrimethamine (as mentioned above). Flow cytometric analysis was performed in 24 hours intervals.

### SYBR green I staining

For each measurement 5 µl of the culture (approximately 800 000 cells) was stained with the DNA-specific dye SYBR green I (Invitrogen, Carlsbad, USA) at 1×. After 20 minutes of incubation, in the dark, the stained sample was immediately analyzed by flow cytometry using a 535/45 nm bandpass filter in front of the detector.

### CD235 (glycophorin A) staining

A volume of 10 µL of a continuous *P. falciparum* culture, at 5% hematocrit, was transferred into a well of a 96 well plate, washed in cold FACS buffer (PBS 1× and 2% bovine albumin serum) and then centrifuged at 1400 rpm, for 3 minutes at 4°C. A volume of 50 µL of a 1∶500 dilution of CD235-Phycoerythrin antibody (eBioscience, San Diego, US) was added to the cells and incubated for 20 minutes on ice in the dark. After a final wash, the cells were re-suspended in PBS 1× and analyzed by flow cytometry using a 610 nm long-pass filter.

### Flow cytometric analysis

**Figure 1:**
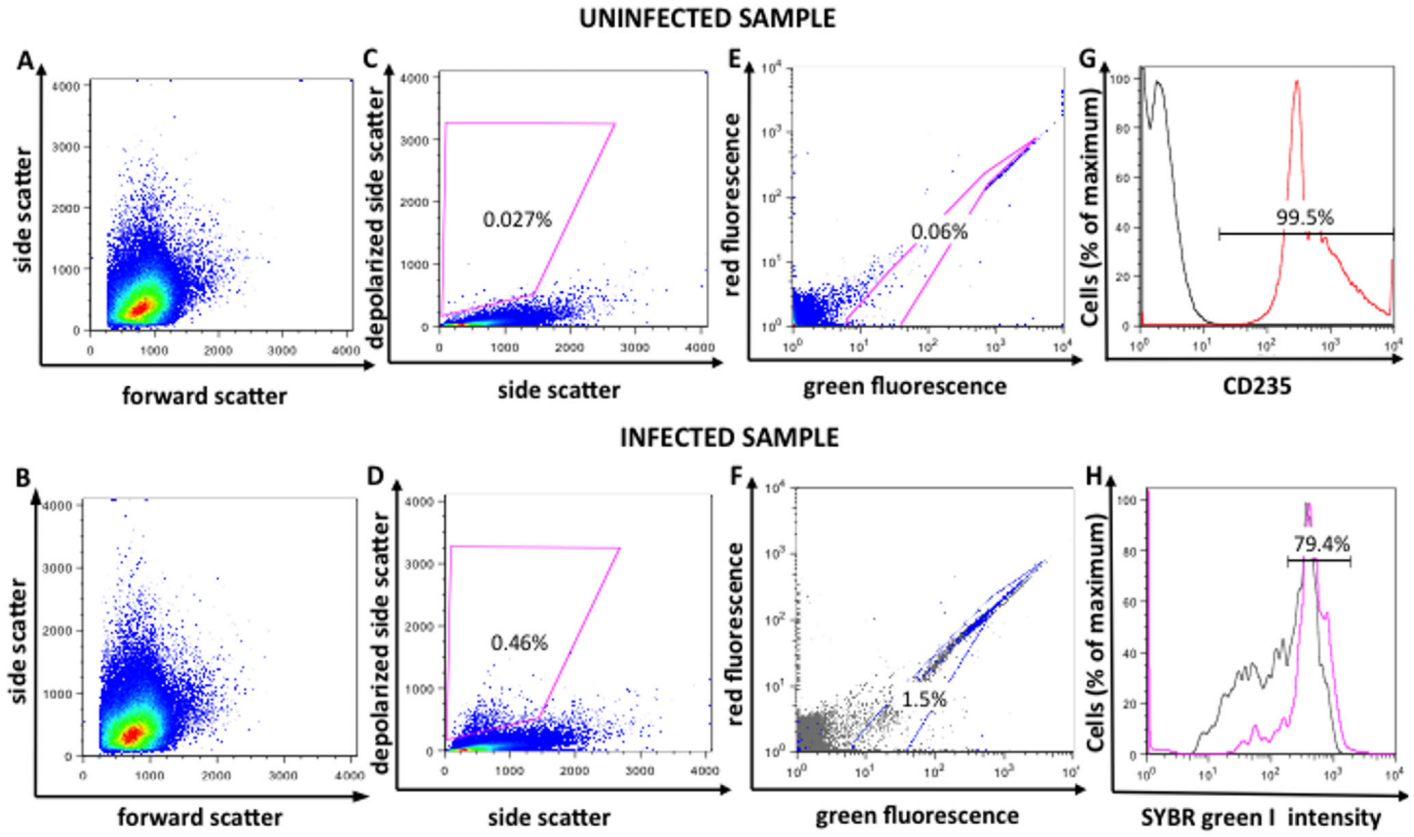
Gating for detection of depolarizing parasitized red blood cells in a *Plasmodium falciparum* culture. Flow cytometric analysis of an uninfected and a synchronized *P. falciparum* (3D7) infected culture (1.5% parasitemia) after 24 hours of incubation, stained with SYBR green I. Plots of forward vs. side scatter for the uninfected and infected cultures appear in Figures A and B; corresponding plots of side scatter vs. depolarized side scatter appear in Figures C and D. The gates in Figures C and D identify the depolarizing events. Figures E and F (see text) illustrate gates defining SYBR green-positive parasitized cells. The blue dots on Figure F represent the depolarizing events. Staining with the red blood cell surface marker (CD235) shows that 99.5% of events in a stained sample (red line) exhibit fluorescence above the highest level measured in an unstained control (black line), indicating that the detected events are red blood cells (G). In the SYBR green I histogram (H) of the infected culture, the overall population (black line) shows a distinct peak with a high fluorescent intensity in the third decade. This peak corresponds mainly to the gated population of depolarizing events (pink line). Because SYBR green I intensity correlates with DNA content and thus with parasite level of maturation, the depolarizing population (pink line) consists mainly (79.4%) of mature parasites. The highly red- and green-fluorescent events visible outside the SYBR green gate just to the right of its apex represent contaminating white blood cells among the donor red cells.

Flow cytometry results were analyzed using FlowJo software (version 9.0.2, Tree Star Inc., Oregon, USA). The gating scheme used is shown in Figure 1. The red blood cells in uninfected and infected samples were detected by their characteristic forward (FSC) and side scatter (SSC) properties (Figure 1A and 1B).

Staining with the anti-glycophorin A CD235 antibody was used to establish that all events detected represented red blood cells. The antibody was not used for routine analyses.

Depolarizing events were defined in plots of SSC versus depolarized-SSC as those with a signal above the background observed in the uninfected control (Figure 1C and 1D).

To determine SYBR green I positive cells, green fluorescence (FL1) versus red fluorescence (FL3) plots were used; these provide better separation between weakly stained and autofluorescent cells than can be obtained from one-dimensional histograms. SYBR green I positive events (Figure 1F) were established based on a stained uninfected control (Figure 1E) and had to be adjusted at each time point, always using the uninfected SYBR green stained sample from the corresponding time point.

### Histidine-rich protein-2 (HRP2) sensitivity assay

A histidine-rich protein-2 (HRP2) enzyme-linked immunosorbent assay (ELISA) was established and performed according to standard procedures [8], also available on the internet website: malaria.farch.net. (http://www.meduniwien.ac.at/user/harald.noedl/malaria/ assessed 3/8/2012)

Ring-stage synchronized *P. falciparum* cultures, at an initial parasitemia of 0.05% and at 1.5% hematocrit, were incubated with the antimalarial drugs for 72 hours, at 37°C in a 5% CO_2_ atmosphere. At the end, samples were frozen at −20°C until the HRP2 ELISA assay was performed.

Briefly, after two freezing and thawing cycles, 100 µL of the lysed sample was transferred to a 96 well-plate pre-coated with MPFM-55A antibody (Immunology Consultants Laboratories, Portland, USA) and incubated for one hour. The cells were washed three times and then incubated for another hour with the secondary antibody, MPFG-55P (Immunology Consultants Laboratories, Portland, USA). Cells were washed again and incubated for 5–10 minutes with the chromogen, TMB One (Biotrend, Köln, Germany). The reaction was stopped by adding sulphuric acid at 1 M (Merck, Darmstadt, Germany) and the absorbance was immediately determined using the Infinite M200 plate reader (Tecan, Männedorf, Switzerland), at a wavelength of 450 nm.

To assess a possible inoculum effect of artemisinin, the HRP2 assay was also performed using a parasitemia of 1%.

### Data analysis

A nonlinear regression model (sigmoidal dose-response/variable slope) was used to calculate the IC50s, with SigmaPlot - Systat Software (Chicago, IL, USA).

## Results

### Depolarized side scatter detects parasitized red blood cells

Staining with the red blood cell surface marker (CD235) antibody was used to establish that the events detected represented red blood cells. Figure 1G shows that 99.5% of events detected are red blood cells. Representative dot plots of side scatter versus depolarized side scatter are shown in Figure 1C and 1D. At 24 hours of incubation, depolarizing events were very low in the uninfected control (0.027%) (Figure 1C), but could easily be detected (0.46%) in the infected blood sample (Figure 1D). The depolarizing events present in the infected sample were also positive for SYBR green I. Analysis of SYBR green I fluorescence intensity showed that the majority of these depolarizing events (79.4%) had high fluorescence (pink line in Figure 1H) indicating high DNA content and thus, represented parasitized red blood cells with mature parasites.

All subsequent results reported in this paper are based on the depolarizing events expressed as a percentage of all events analyzed.

### Depolarized side scatter detects parasite maturation

**Figure 2:**
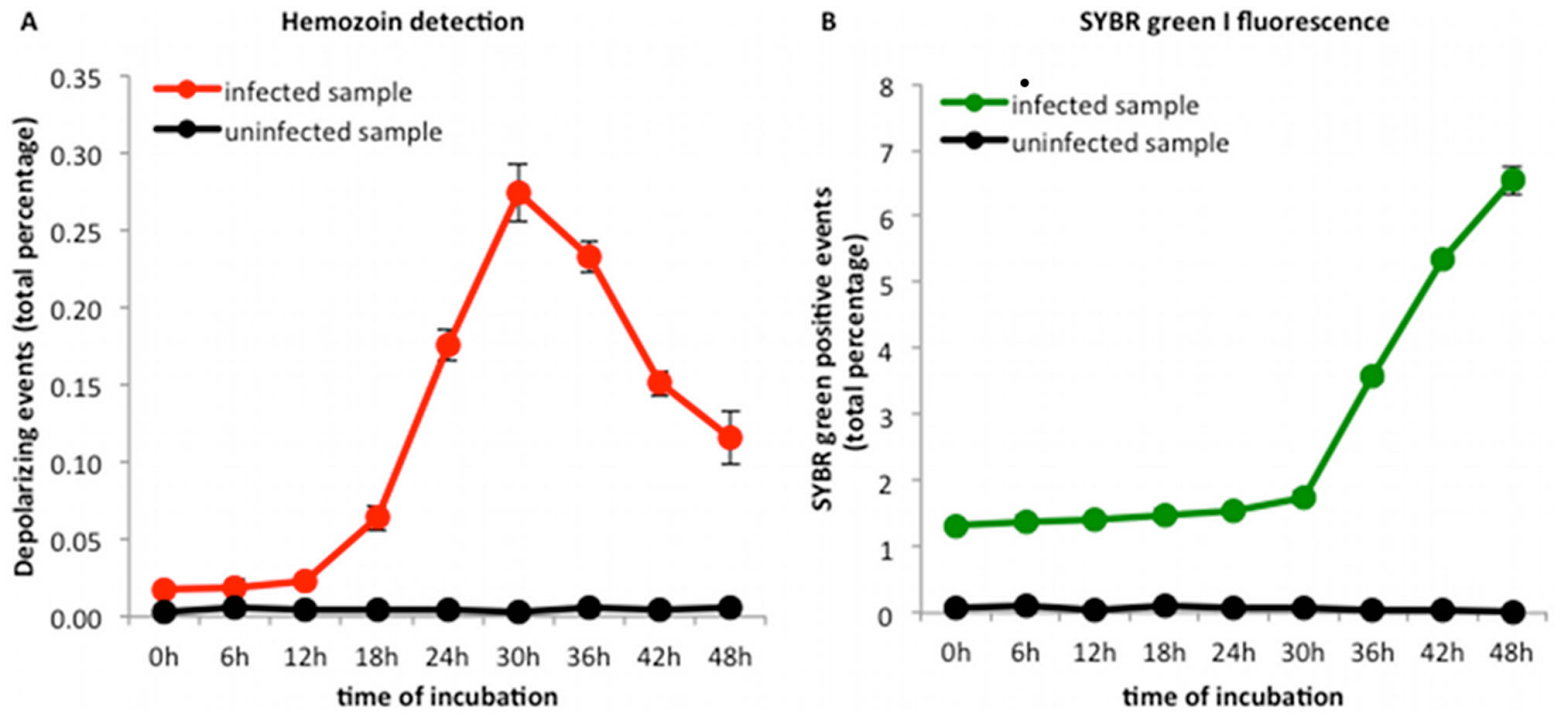
Growth curves of *Plasmodium falciparum* (3D7) in culture. Flow cytometric analysis of a synchronized *P. falciparum* (3D7) culture (1.4% parasitemia). The percentages of depolarizing events (A) and SYBR green I positive events (B) were followed for 48 hours in uninfected (black lines in A and B) and infected red blood cells (red line in A and green line in B). Analysis of depolarizing events (Hz-containing parasitized erythrocytes) shows an increase at 18 hours, peaking at 30 hours (A). SYBR green I positive events (parasitized RBC) remain unchanged at 1.4% until 30 hours, after which a steady increase can be noted (B). Hz detection reflects parasite maturation with increasing amounts of Hz until 30 hours, while the parasitemia remains unchanged (SYBR green I positive events). After 30 hours, increasing SYBR green I positive events indicate replication and presence of immature forms, which explains the decrease observed in the depolarizing population. Each time point represents the mean value of triplicate samples ± one SD. Red blood cell lysis was excluded by absolute cell counts, which remained stable.

We next sought to establish whether this type of analysis would discriminate among different *Plasmodium falciparum* stages. To that end, samples of 100,000 events were analyzed. Determination of the absolute number of cells at each time point showed no evidence for red blood cell lysis. Following the percentage of depolarizing events during 48 hours of incubation, in a ring-stage synchronized culture (1.4% parasitemia), showed an increase at 18 hours, with a peak at 30 hours and a subsequent decrease (Figure 2A). The same sample stained with the DNA stain, SYBR green I, showed no change in the percentage of fluorescent events until 30 hours, followed by a steady increase until 48 hours (Figure 2B). Observation of Giemsa stained blood smears at all time points showed parasite maturation until 30 hours, which coincided with the peak of depolarizing events. Thereafter, from 36 to 48 hours, immature forms were observed, coinciding with a decrease of depolarizing events and an increase of fluorescent events (Figure 2). These changes reflect parasite growth with the 30 hour peak of depolarization corresponding with the peak of maturation, after which erythrocytes rupture and daughter-merozoites are released along with Hz, explaining the decrease in depolarizing events and the increase in SYBR green I fluorescent events, observed after 30 hours.

The increase in the percentage of depolarizing events over time and the inhibitory effect of chloroquine, artesunate and pyrimethamine could be detected at parasitemias down to 0.3%. At lower parasitemias (0.05 and 0.1%) no clear increase above the background could be detected during the first 48 hours of incubation.

### Detection of inhibitory effects of chloroquine on sensitive (3D7) and resistant (Dd2) *P. falciparum* strains

**Figure 3:**
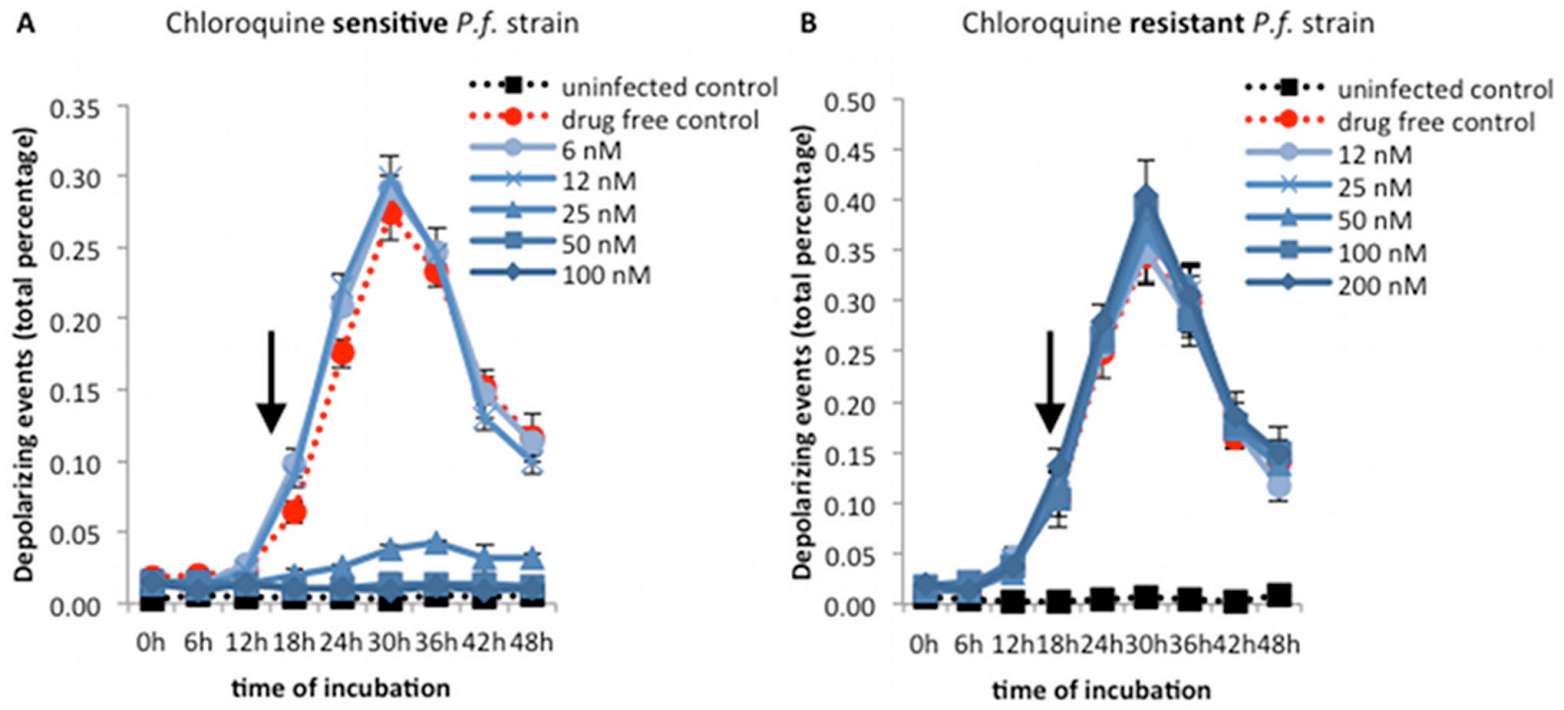
Effect of chloroquine on the growth curve of *P. falciparum* sensitive and resistant strains. Synchronous cultures of sensitive (3D7, parasitemia of 1.3%) and resistant (Dd2, parasitemia of 1.4%) *P. falciparum* strains were incubated for 48 hours with doubling concentrations of chloroquine and analyzed at 6 hourly intervals. The inhibitory effect of chloroquine at higher concentrations (>25 nM) is clearly visible (arrow) after 18 hours of incubation (A). The resistant strain can easily be distinguished from the sensitive strain with growth curves of all drug concentrations being identical to the drug free control (B). Each time point represents the mean value of triplicate samples ± one SD.

Using a chloroquine sensitive strain (3D7), the difference between inhibiting and non-inhibiting concentrations was clearly visible after 18 hours, with the largest difference observed at 30 hours (Figure 3A). The first sign of drug effect could be consistently detected at 18 hours, where at concentrations of 6 and 12 nM a percentage of 0.1% depolarizing events were observed, while at 25, 50 and 100 nM only 0.004% were detected (Figure 3A).

In the resistant *P. falciparum* strain (Dd2), chloroquine resistance could clearly be detected at 18 hours after drug exposure, with growth curves of all concentrations following the drug free control (Figure 3B). At this time point (18 hours), in all drug concentrations around 0.1% depolarizing events were observed, compared to the 0.01% seen at the beginning of the incubation (Figure 3B).

### Detection of inhibitory effects of other antimalarial drugs

**Figure 4:**
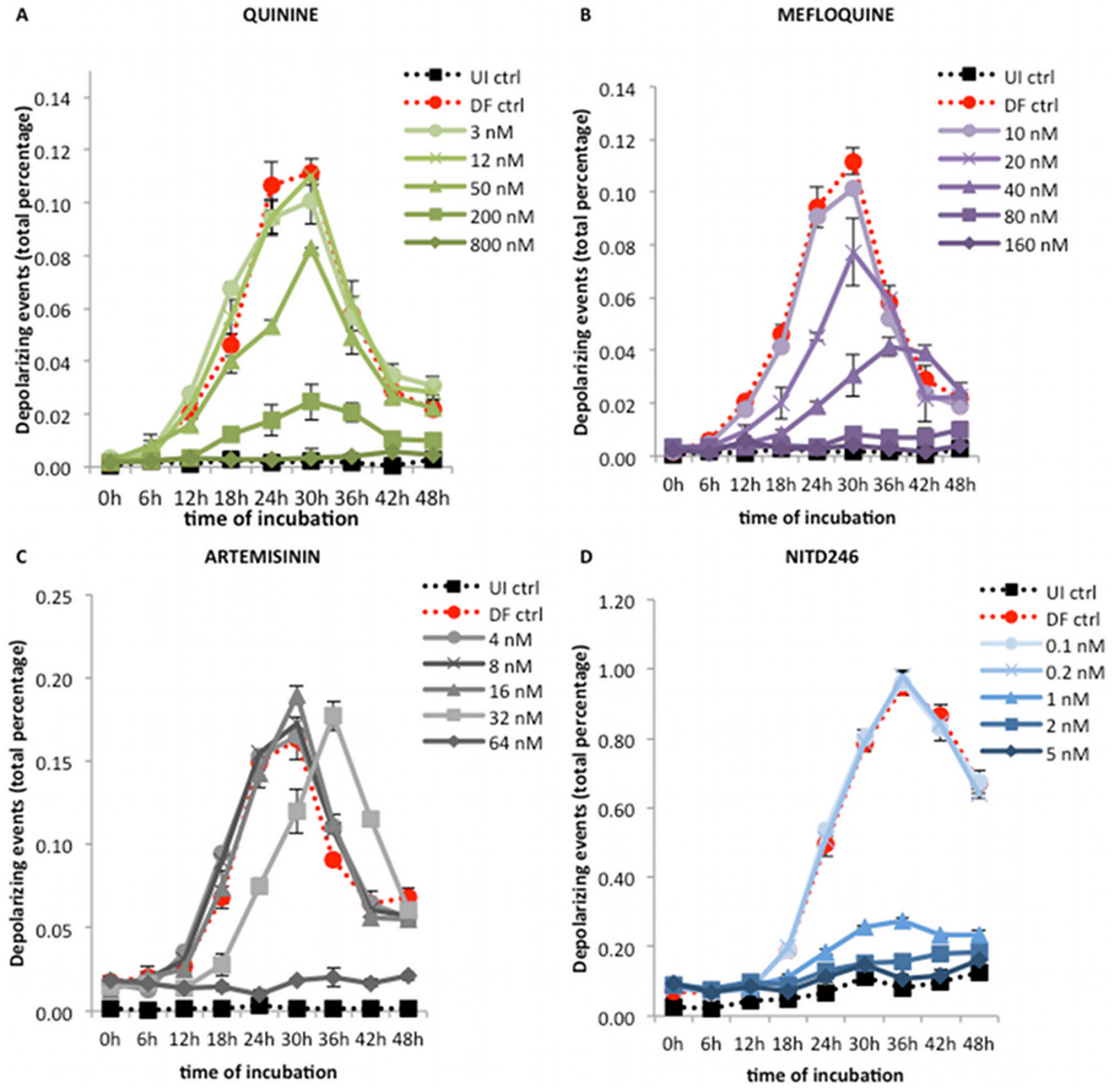
Effect of quinine, mefloquine, artemisinin and a spiroindolone (NITD246) on the growth curve of *P. falciparum* (3D7). Synchronous cultures of a *P. falciparum* 3D7 strain were incubated for 48 hours with increasing concentrations of quinine (A), mefloquine (B), artemisinin (C) and NITD246 (D). In all cases the dose-dependent inhibitory effect of the drugs could already be detected at 18 hours by comparing the treated samples (solid lines) with the drug free control (dotted red line). The curves allowed the determination of IC50 values at 24 hours. Of note, artemisinin at 32 nM showed a 6 hour delayed growth curve, from 18 to 42 hours, with the peak of maturation occurring at 36 hours. Each time point represents the mean value of triplicate samples ± one SD. DF ctrl – drug free control; UI ctrl – uninfected control.

**Figure 5:**
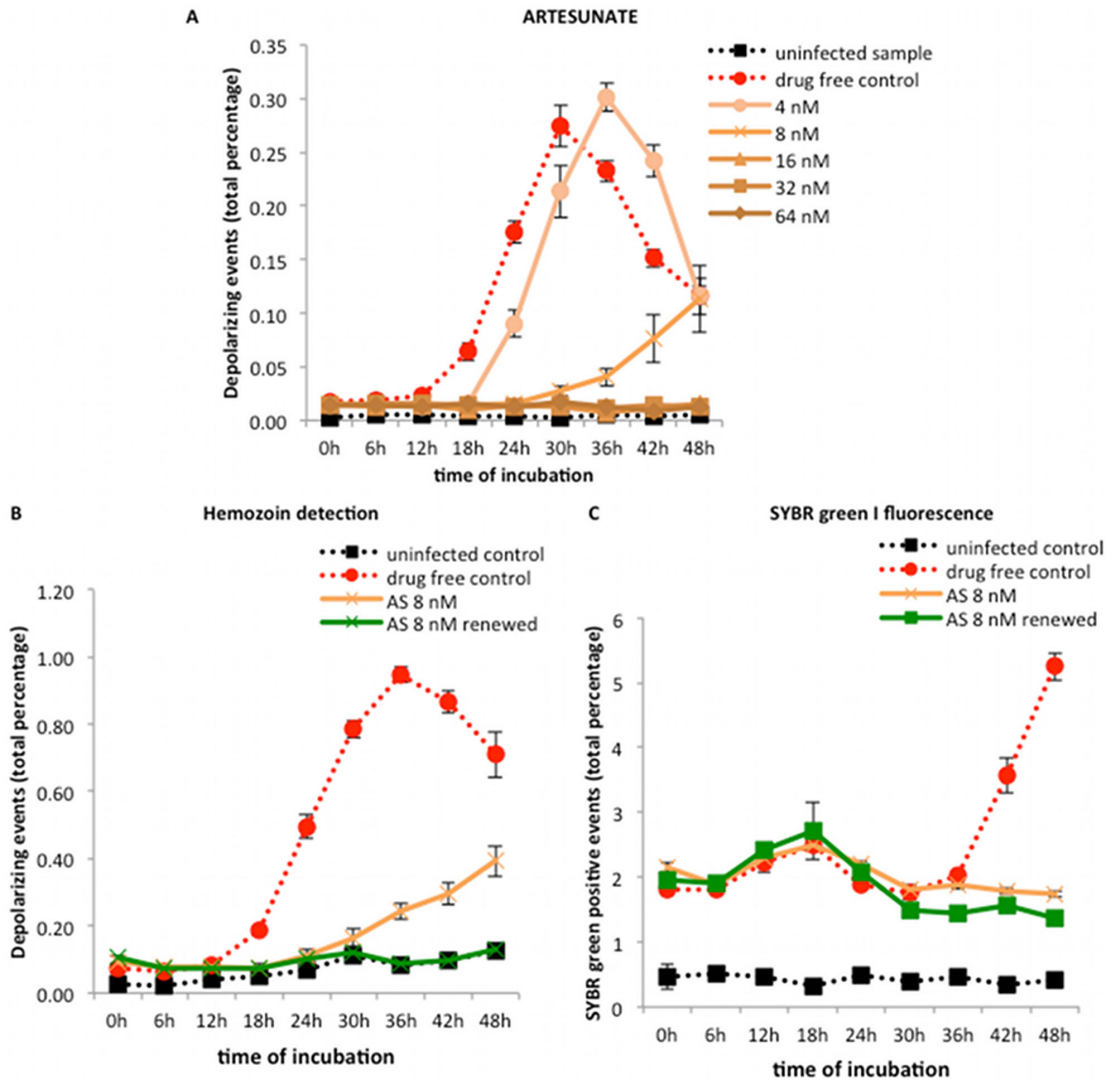
Effect of artesunate on the growth curve of *P. falciparum* (3D7) and the effect of 12 hourly renewing of artesunate during incubation. Synchronous cultures of a *P. falciparum* 3D7 strain were incubated for 48 hours with doubling concentrations of artesunate (A) or with a single concentration of 8 nM of artesunate for the whole time or renewed at 12 hour intervals (B and C). Figures A and B show detection of Hz (depolarizing events) while Figure C shows detection of SYBR green I fluorescence (DNA in parasites). The inhibitory effect of artesunate was already detectable after 18 hours of incubation (A). Similar to artemisinin (Figure 4C), the growth curve of artesunate at 4 nM showed a 6 hourly delayed growth curve from 18 to 42 hours, including a 6 hour delay in the peak, occurring at 36 hours. Interestingly, the growth curve at 8 nM seemed to show inhibition until 30 hours, when a slight increase was observed (A and B). However, renewing artesunate at 12 hourly intervals eliminates this effect (green line in B). The percentage of SYBR green I positive events remained approximately the same during the 48 hours of incubation (C). This indicates that parasites at the non-renewed 8 nM concentration showed some maturation as indicated by Hz detection but were unable to replicate. Each time point represents the mean value of triplicate samples ± one SD.

**Figure 6:**
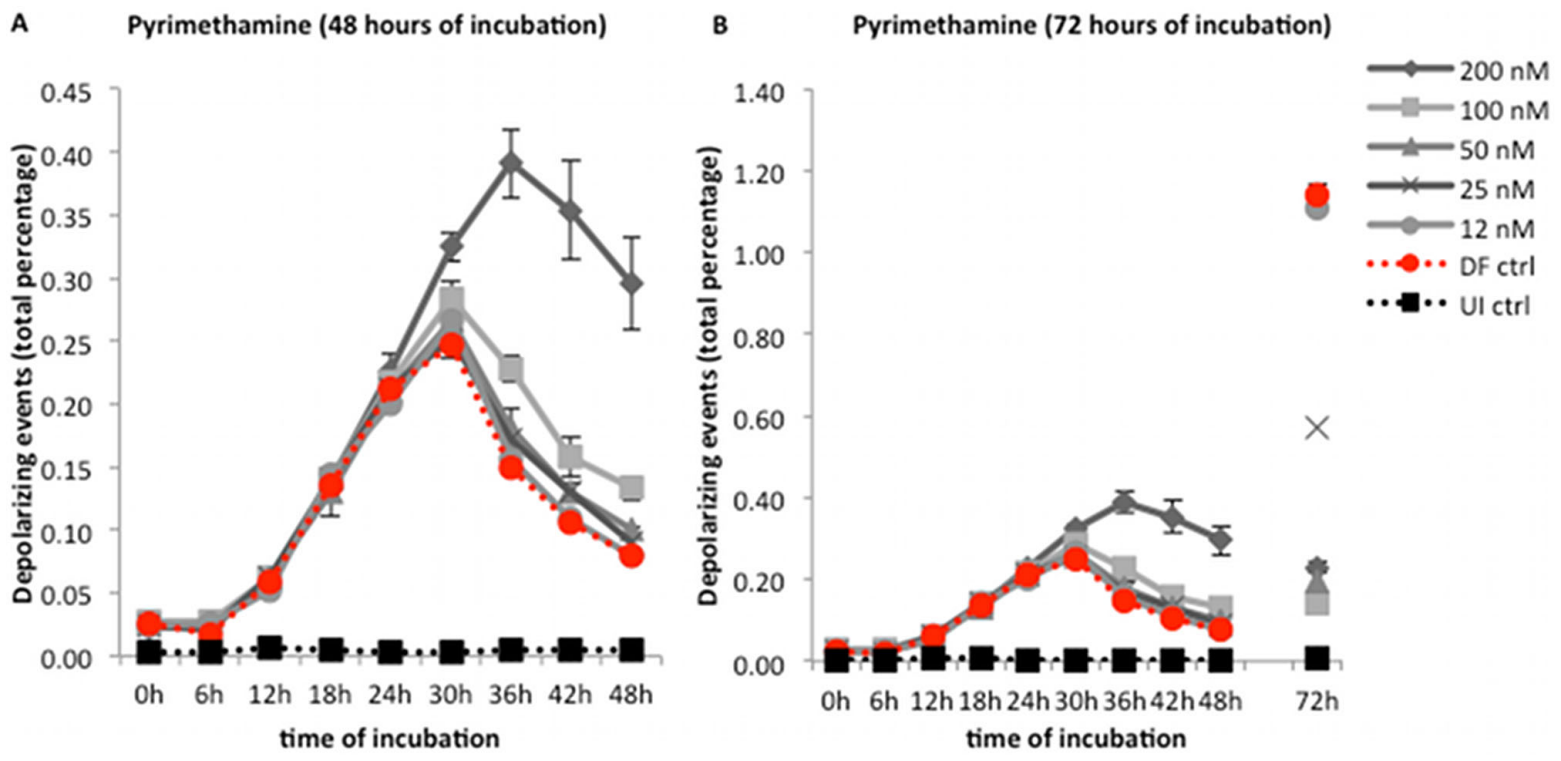
Growth curve of *Plasmodium falciparum* (3D7) after treatment with pyrimethamine. Synchronous cultures of a *P. falciparum* 3D7 strain were incubated for 72 hours with doubling concentrations of pyrimethamine. No inhibitory effect could be observed during the first 48 hours at any concentration (A). However, an inhibition was clearly visible at 72 hours (B). This could be explained by the fact that pyrimethamine is a slow acting drug and its effect can only be detected on the second generation, after 48 hours. Interestingly, the growth curves at all concentrations follow the drug free control after the 30 hour peak, while the curve for 200 nM shows a later peak at 36 hours with a higher number of depolarizing events, compared to the drug free control (A). See discussion for possible explanation. Each time point represents the mean value of triplicate samples ± one SD. DF ctrl – drug free control; UI ctrl – uninfected control.

Representative growth curves in the presence of different concentrations of quinine, mefloquine, artemisinin and the spiroindolone NITD246 are shown in Figure 4. The curves for artesunate are shown in Figure 5 and those for the slow acting drug, pyrimethamine, are shown in Figure 6. As was the case with chloroquine, inhibitory concentrations showed a clear effect from 18 hours onwards in three different compound classes: quinolones (quinine and mefloquine), endoperoxides (artemisinin and artesunate) and a spiroindolone (NITD246).

Interestingly, artemisinin showed a delayed growth curve at the 32 nM concentration in comparison to the drug free control (Figure 4C). The same was observed for artesunate at a lower concentration of 4 nM (Figure 5A). Of note, the artesunate growth curve at an intermediate concentration of 8 nM showed an initial inhibition with a delayed rise starting after 30 hours and an absence of the typical peak at 24–30 hours (Figure 5A and B). By renewing the artesunate every 12 hours in the culture medium, this delayed rise was lost and the initial inhibition was maintained throughout the 48 hours of incubation (Figure 5B). In both cases, however, the percentage of SYBR green I positive events remained largely unchanged over the 48 hours of incubation, indicating absence of parasite replication (Figure 5C). The parasites, previously treated only once with artesunate at 8 nM, were also re-cultured and observed for growth during four days. No growth was detected during these four days, as confirmed by flow cytometry, nor was an increase in either depolarization or SYBR green percentage and intensity observed.

### Inhibitory effect of slow-acting drugs (pyrimethamine)

Pyrimethamine (Figure 6A and B) required an incubation time of 72 hours to reliably detect drug effects (Figure 6B). Interestingly, while concentrations of 12 to 50 nM followed the drug free control, the curve of 100 nM showed slightly higher values from 30–48 hours, while the highest concentration of 200 nM showed a marked increase of depolarizing events and a delayed peak at 36 hours, with twice as many events (Figure 6A).

### IC50 values obtained by the Hemozoin detection assay are comparable with other available assays

To determine the earliest time-point that would allow us to reliably calculate IC50 values, results obtained from the Hz detection assay at different time-points (18, 24, 30 and 36 hours) (Table 1) were compared with those reported in the literature (Table S2), as well as with results from the already validated HRP2 ELISA assay (Table 2). This analysis led us to use the 24 hour time point for all subsequent IC50 calculations. The IC50 results for our Hz assay and the HRP2 assay are shown in Table 2.

**Table 1:** Inhibitory concentrations (50%) of several antimalarial drugs against *P. falciparum* 3D7 strain determined by the Hemozoin detection assay at different times of incubation.

	Time of incubation				
	18 h*	24 h	30 h*	36 h*	72 h*
**Chloroquine**	41.9 nM	34.2 nM	33.6 nM	35.9 nM	29.6 nM
	(±19.3)	(±8.1)	(±10.1)	(±10.1)	(±7.4)
**Quinine**	98.5 nM	54.6 nM	92.2 nM	142.7 nM	45 nM
		(±9)			
**Mefloquine**	17.2 nM	21.3 nM	33.5 nM	64.9 nM	19.5 nM
		(±7)	(±6.4)	(±20.2)	
**Artemisinin**	28.2 nM	25.6 nM	34.5 nM	43.7 nM	n.d.
	(±3.5)	(±5.9)	(±5.8)	(±16)	
**Artesunate**	< 4 nM	6.4 nM	10.8 nM	12.4 nM	n.d.
		(±2.3)	(±3.6)	(±6.3)	
**Pyrimethamine**	X	X	X	X	25.4 nM
					(±10.3)

Mean inhibitory concentration values (50%) ± one standard deviation are presented.

Standard deviation values are not shown for results that were not supported by at least three independent experiments. Time-points identified with (*) were not systematically analyzed, since the 24 hour time-point was used as the preferential time-point to reliably calculate IC50 values (as discussed in the manuscript).

(X) values could not be determined; (n.d.) no data available.

**Table 2:** Antimalarial activities of several antimalarial drugs determined by the Hemozoin detection assay and the HRP2 assay.

	Hemozoin detection* 24 hours of incubation	HRP2 72 hours of incubation
**Chloroquine**	34.2 nM (±8.1)	22 nM
**Quinine**	54.6 nM (±9)	52 nM
**Mefloquine**	21.3 nM (±7)	21 nM
**Artesunate**	6.4 nM (±2.3)	1.1 nM
**Artemisinin**	25.6 nM (±5.9)	11.5 nM
**Pyrimethamine**	25.4 nM (±10.3)1)	30.5 nM
**NITD 246**	0.8 nM	0.4 nM

The averages of 50% inhibitory concentration values ± one standard deviation are presented above.

* For the Hemozoin detection assay each drug was tested at least three times (except for the novel compound NITD246).

HRP2 – Histidine-rich protein 2;

1) after 72 hours of incubation.

Concerning the inoculum effect, no reliable results could be obtained with the HRP2 assay at a parasitemia of 1%, because no differences were observed between the drug treated samples and the drug free control. Using a lower parasitemia in the Hz assay, discrepant results were observed for artemisinin and artesunate: while the IC50 of artesunate remained the same (4 nM) at a parasitemia of 1.3% and 0.4%, the IC50 value for artemisinin decreased from 32 nM at 1.0% parasitemia to 13.2 nM at a parasitemia of 0.7%.

## Discussion

This study confirms that the optical detection of Hz can be easily achieved by a simple adaptation of a common flow cytometer to allow detection of light depolarization (Figure S1). This study also extends observations [27] that maturation of *P. falciparum* and inhibitory antimalarial drug effects can be assessed by the detection of Hz inside intra-erythrocytic parasites. Although this novel approach is based on previous observations that Hz in parasitized RBC could be detected by flow cytometric methods [24], [27] it should be noted that the idea of using Hz for a sensitivity assay is not new and was described in the 1980s [31]. However, the assay format used a visual readout and appears to have been rather inaccurate because of nonspecific agglutination [32]. Recently, an improvement of this approach has been reported where the Hz produced is measured by a colorimetric method [33]. To do this, the Hz produced by the parasites after 72 hours of incubation is liberated and transformed back into heme before reading the absorbance at 405/750 nm. However, the assay involves multiple manipulation steps, including lysis and several washing steps, which are cumbersome and may introduce variability. In fact, in our hands, the isolation of Hz and quantification of heme requires meticulous attention to accurate pipetting to guarantee reproducible results [34].

### Depolarization signal strength and detection limit

The comparison of an uninfected RBC sample with a synchronized *P. falciparum* infected RBC sample at 24 hours of incubation showed a depolarizing population that could be easily identified and gated (Figure 1D). However, the degree of depolarization of the whole population was much lower than previously described for *P. berghei* infected blood samples [27]. A possible explanation for this may be different side scatter signals, which are a measure of cell granularity [35], that could be caused by the different shape and distribution of the Hz crystals within these parasites. A parasite containing several small but distributed Hz crystals will have a higher depolarized side scatter signal than a parasite containing a single big Hz crystal. In fact, during this and previous work, the routine microscopic analysis of Giemsa stained blood smears to control the parasitemia showed that Hz started to appear in small dispersed fine granules in *P. berghei* and only clumped together by the end of schizogony, as previously observed by Warhurst et al. [36], while in *P. falciparum*, Hz crystals aggregate as they start to appear (data not shown). It also appears possible that the depolarization signal can still be improved as a result of technical modifications to the instrument. The Cyflow® flow cytometer uses a blue laser (488 nm). However, longer wavelengths may increase the depolarization signal as has been described for a red HeNe (633 nm) or Kr^+^ laser (647 nm) [18]. Furthermore, the polarization ratio of most solid-state lasers is usually given as >1∶100. If the laser had a higher polarization ratio, as do HeNe and Kr+ lasers, positive depolarization signals might be better distinguished from background noise.

To assess parasite maturation and drug effects we chose to use the simple ratio of all identifiable depolarizing events as a percentage of all events analyzed (Figure 1C and D). Other measurements, like depolarizing intensity, showed no clear advantage (data not shown). A parasitemia of 1% proved to be the optimal parasitemia to detect parasite maturation and growth over 48 hours. Thus, it was used in the Hz detection assay to obtain time-curves when investigating the drug effects in *P. falciparum in vitro* cultures. Still, an initial 1% parasitemia is higher than the ones used in other assays, such as the HRP2 or the WHO schizont maturation test, which can use parasitemias as low as 0.02% [8], [37], [38]. However, when investigating the lower detection limit of the Hz assay, parasite maturation and drug effects of chloroquine, artesunate and pyrimethamine could still be clearly detected at parasitemias as low as 0.3%. Nevertheless, this remains higher than the ones used for the HRP2 assay and the WHO schizont maturation test. Yet, it is comparable to the parasitemias used in other assays, such as the [3H]-hypoxanthine assay (0.25–0.5%) [6] or the SYBR green I assay (0.5–1%) [9], [39], [40] (Table S1).

Of note, in all Hz assay experiments, only around 30% of all parasites were typically detected by depolarization measurements (Figure 1D) as compared to microscopy or SYBR green I fluorescence. The reasons for this are unclear and could be a consequence of the culture not being highly synchronized. At the 30 hour peak of maturation, schizonts as well as some second generation ring forms are present, as confirmed by microscopy; the immature ring forms have insufficient Hz to be reliably detected [28]. Another possible explanation is the already mentioned Hz aggregation that occurs in the mature parasites, which may reduce side scatter intensity.

Certainly, the initial parasitemia of 0.3% required is still a major limitation of this assay if used directly with patient blood samples, some of which may have lower parasitemias [41]. However, further optimization of the assay may lead to an improvement of the detection limit. Nevertheless, future studies conducted in the field will allow us to evaluate the performance of the novel Hz detection assay using *ex vivo* patient samples.

### Aspects of parasite maturation

Using a red blood cell surface marker (CD235) (Figure 1G) and a DNA stain (SYBR green I) we showed that the depolarizing events were indeed infected red blood cells (Figure 1F). SYBR green I fluorescent intensity reflects DNA content [42], [43]. Most of the depolarizing events showed high SYBR green I fluorescence (80%), indicating that they were mature parasites (Figure 1H). Comparing depolarization and fluorescence intensity in a *P. berghei* ANKA infected sample stained with SYBR green I showed, similarly, that the level of depolarization appears to reflect parasite maturation [27].

Usually, the described *P. falciparum* life cycle lasts 42–48 hours *in vitro* [44]. Contrary to this, we observed a peak of parasite maturation earlier, at around 30 hours, as reflected by the peak in depolarization (Figure 2A) followed by a steady increase in SYBR green I positive events indicating replication (Figure 2B). Corresponding parasite forms were also observed during microscopic observation of Giemsa stained blood smears. This can be explained by the fact that because the assay did not start immediately after re-invasion thus, the indicated time points correspond to the time post-drug-treatment and not the time post-invasion. Moreover, the fact that cultures were not highly synchronized seem to have contributed to this apparent shorter life cycle. It is known that to obtain highly synchronized cultures, at least one other sorbitol treatment would be needed [45].

After a single synchronization approximately 90% of the parasites are ring forms. However, individual parasites can present differences of several hours in their development. Thus, at the beginning of the experiments, some parasites may have already developed for 12 hours within the ring form population while others are only 6 hours into their development. The microscopic observation of Giemsa stained blood smears at 36 hours gives weight to this reasoning, because both schizonts and second generation ring forms could be observed at this time point.

### Drug effects and potential use of the Hz assay

One of the major advantages of the Hz detection assay is the fact that samples can be easily analyzed without further preparation or additional reagents allowing a rapid and easy real-time assessment of parasite maturation and drug effects. For instance other assays, such as the [3H]-hypoxanthine assay, cultures have to be incubated for additional 24 hours with the isotope before measurements are possible [6], [15], [46]–[48]. Another advantage is the early detection of inhibitory drug effects after only 18 hours (Table 1 and Figures 3, 4 and 5), in contrast to several other assays, such as the [3H]-hypoxanthine assay [6], [48], which measures drug effects at 48 hours, the SYBR green I [15], [40], [49], [50] and the HRP2 [8], [15], [37] assays, which require at least 72 hours.

Although, for example, in the Hz assay chloroquine sensitive and resistant strains are easily distinguishable after only 18 hours (Figure 3), the inhibitory effect of the slow acting drug pyrimethamine could only be detected after 72 hours (Figure 6). This drug has no effect on asexual parasites in the first half of the parasite life cycle (24 hours) [41], [51] and effects can only be detected later in the second parasite generation. For this reason the Hz assay detects slow acting drugs effects, such as pyrimethamine, only after 72 hours of incubation. Interestingly, at concentrations of 200 nM, higher depolarization values were observed after 30 hours (Figure 6A), which appear to reflect schizont arrest, which was observed by microscopy.

As shown in Table 1, the Hz detection assay allows determination of a drug's inhibitory concentrations at different time-points during the first and second life cycle, thus extending its potential usefulness.

### Artemisinins in the Hz assay

Artemisinin and artesunate growth curves were different from the other tested drugs. At concentrations of 32 nM and 4 nM, respectively, a delayed maturation was observed, starting at 18 hours (Figures 4C and 5A). However, the SYBR green I measurements performed simultaneously indicated that the parasites replicate, reaching approximately the same parasitemia as the drug free control after 48 hours of incubation (data not shown). The reason for this observation remains unclear. Furthermore, samples treated with 8 nM of artesunate showed an initial inhibition with a delayed rise after 30 hours (Figures 5A and 5B). SYBR green I measurements, however, did not change over the 48 hour incubation period, indicating that the parasites were somehow able to mature but unable to replicate (Figure 5C). These parasites, treated a single time with 8 nM of artesunate, were re-cultured in CMCM and no growth could be observed during the following four days. Interestingly, similar behavior has been previously described for artemisinin [52] and was interpreted as a consequence of its rapid degradation [53]. The drug level had to be kept constant by replacing the media with the drug every 24 hours, otherwise the number of viable parasites increased at 96 and 120 hours after drug addition [52]. In fact, renewing the artesunate every 12 hours in the culture medium showed that the inhibition was maintained throughout the 48 hours of incubation and the delayed rise was lost (Figure 5B).

Dormancy has been described after treatment with artemisinins, where early stage parasites enter a dormant stage under drug pressure but later regrow [54]. It has been reported that parasites treated with dihydroartemisinin arrested their development shortly after drug exposure, but after 9 days 50% of the parasites managed to resume their growth [54]. Thus, to detect recrudescence of these dormant parasites cultures would have to be monitored for at least four life cycles (9 days = 216 hours). Our findings showed a delayed increase during the first life cycle (after 30 hours of incubation), in samples treated with artesunate at 8 nM. Although this might represent the detection of dormancy and parasite recrudescence, the data we have obtained so far are not sufficient to confirm or exclude this hypothesis, because these parasites were not monitored for longer than four days. Further investigation is needed to clarify this issue.

### Comparison of IC50 values

The IC50 determined by the Hz detection assay were comparable with the ones reported for other, already validated assays (Tables 1, 2 and S2). However, slightly higher values were observed for some of the drugs, especially for artesunate, where most publications report values below 2.5 nM [15], [37], [47]. One explanation could be the inoculum effect, i.e., an increase in the inhibitory drug concentration when greater numbers of parasites are inoculated. This is thought to be the consequence of some drug accumulating inside the parasitized RBC [55] and has been described for drugs like artemisinin and artesunate, as well as for chloroquine and mefloquine [56]. In fact, in the Hz assay parasitemias of around 1% were used as compared to other assays, which used parasitemias ranging from 0.05 to 0.5% [6]–[9], [37]–[40]. This might explain the increased IC50 values observed in the Hz detection assay and interestingly, a decrease in the parasitemia from 1.4% to 0.7% led to a decrease in the artemisinin IC50 value from 32 to 13.2 nM. However, no such decrease was observed with artesunate.

It is important to note, that the comparison of IC50 values between assays can be misleading. The IC50 values in the literature vary substantially between assays, even for the same strain. For instances, the standard [3H]-hypoxanthine incorporation assay shows differences of 2 to 23 fold for artesunate (Table S2). Not too surprisingly, one study reported that the reliability of the assays may be influenced by the mechanism of action of individual drugs [15]. Moreover, variations in parasite density and hematocrit as well as the stage-dependent action of antimalarial drugs, may have a significant impact on the outcome of these sensitivity assays [14], [56].

### Assay – prospects, future possibilities

Because the Hz detection assay allows parasite maturation to be monitored in real time, it could possibly be used to investigate drug effects on different parasite developmental stages (stage specificity of drugs). It might also be interesting to assess the performance of the assay with parasite strains that have been reported to show clinical signs of resistance to artemisinins. Since modeling of parasite-clearance curves suggests that artemisinin resistance affects ring-stage parasites more than the more mature parasite stages, thus *in vitro* tests focusing on the inhibition of ring-stage parasites could become valuable surveillance tools [57].

The fact that a flow cytometer is required may pose an obstacle to the widespread use of the Hz assay in the field. Furthermore, flow cytometers that use fiber optic cables for light collection, such as the BD LSRFortessa or FACSaria, require relatively elaborate optical modification to detect depolarization. However, detection of depolarization as we have described can be implemented simply on many existing flow cytometers, for example the flow sorter Influx (http://www.bdbiosciences.com/instruments/influx/features/index.jsp, accessed 14/08/2012), the Cyflow Cube (Danny Koehler, Partec, Münster, Germany, personal communication), the Life Technologies Attune (Grace Chojnowski, Queensland Institute of Medical Research, Brisbane, Australia, personal communication), and can even be retrofitted to the venerable BD FACSCalibur (Lisa Nichols, Cytek Development, Fremont, CA, USA, personal communication). More importantly, some low-cost flow cytometers for CD4 counts in resource-poor countries could be modified rather easily to detect depolarization, and are already available on site in several African countries (http://www.partecnorthamerica.com/Press-Release-01Dec2012-_b_2.html assessed 22/12/12).

Furthermore, it is now evident [58] that optical measurements of both DNA and Hz can be made in small, robust, simple, widefield multiparameter optical imaging apparatus an order of magnitude less expensive than a typical flow cytometer, using LEDs costing only a few dollars for illumination and employing camera chips only slightly higher in quality than those used in mobile phones as detectors. Signals from all cells in an entire well of an assay microplate can be analyzed in seconds, without the need for precise stage motion or focus adjustment. Somewhat more elaborate and expensive versions of such devices are already commercially available; minimalist instruments optimized for field use in the resource-poor areas in which malaria is prevalent should arrive within a few years.

In conclusion, the novel Hz detection assay allows parasite maturation to be monitored in real time without the need of further reagents or sample preparation. The assay detects inhibitory drug effects of major antimalarial drug classes after only 18 hours of incubation and permits determination of IC50 values at 24 hours. Future work will have to address the utility of this assay in the field. Issues such as the use of less expensive alternatives to flow cytometry and the application of the assay to such tasks as the determination of stage specific effects of antimalarial drugs will have to be investigated further.

## Supporting Information

Table S1 **Comparative descriptions of available** ***in vitro*** **sensitivity assays for** ***Plasmodium falciparum.***
(DOCX)

Table S2 **Antimalarial activities of several antimalarial drugs determined by different** ***in vitro*** **sensitivity assays against** ***P. falciparum*** **3D7 strain.**
(DOCX)

Figure S1 **Cyflow® flow cytometer and optical bench layout.** Images in the top row show the components (A) and the optical bench layout (B) of the Cyflow® flow cytometer. The 488 nm laser light is vertically polarized. A horizontally polarized filter is placed in front of a second side scatter detector to allow detection of depolarized light (depol SSC). Images C, D and E show a Cyflow® in the laboratory and being easily packed for transport at the Centre de Recherches Médicales de Lambaréné – CERMEL, Lambaréné, Gabon. (The subject of the photograph has given written informed consent, as outlined in the PLOS consent form, to publication of the photograph).
(DOCX)
