# Supplementary material for: A Novel Flow Cytometric Hemozoin Detection Assay for Real-Time Sensitivity Testing of Plasmodium falciparum
Source: PLoS One. 2013 Apr 24;8(4):e61606. doi: 10.1371/journal.pone.0061606 (PMC3634823; doi:10.1371/journal.pone.0061606)
Supplement: Table S1 — Comparative descriptions of available in vitro sensitivity assays for Plasmodium falciparum. (DOCX) [file pone.0061606.s001.docx]

|  | **Microtest** | **[3]-Hypoxanthine incorporation** | **Flow cytometric assays** | **Fluorometric assays** | **HRP2 assay** |
| --- | --- | --- | --- | --- | --- |
| **Principle of the assay** | Quantification of schizonts | Measurement of parasite DNA | | | Antigen detection |
| **Time of incubation with the drugs** | 30h | 48h | 48h | 48 - 96h | 72h |
| **Volume of sample to analyze** | 10 – 50 μL | 200 μL | 25 - 100 μL | 10 – 200 μL | 100 μL |
| **Hematocrit** | 1.5 – 20% | 1 – 2.5% | 2.5% | 1.5 – 2.5% | 1.5% |
| **Parasitemias:** |  |  |  |  |  |
| **Laboratory strains** | 1% | 0.5% | 0.25 – 1% | 0.5 -1% | 0.05% |
| **Clinical isolates** | 0.02% | 0.1-0.5% | (n.d.) | 0.5 - 1% | 0.05% |
| **Required equipment and Infrastructure** | Bright-field microscope | Scintillation counters and harvesting machine and infrastructure | Flow cytometer | Microplate fluorometer | Microplate spectrophotometer |
| **Advantages** | - Requires little equipment. | - Automatic reading of results. | - Automatic reading of results. | - Simple, does not require specialized personnel. | - Simple, does not require specialized personnel. |
|  |  | - Accurate and sensitive. | - Accurate and sensitive. | - Accurate. | - Low detection limit (0.05% parasitemias). |
| **Limitations** | - Subjective and labor-intensive. | - Costly equipment. | - Costly equipment and reagents (e.g.: DNA stains). | - Costly reagents (e.g.: DNA stains). | - Costly reagents (e.g.: antibodies). |
|  | - Highly trained personnel. | - Handling of radioactive reagents. | - Additional incubation steps with reagents. | - Additional incubation steps with reagents. | - Additional incubation steps with reagents. |
|  |  | - Additional incubation steps with reagents. |  |  |  |

**Table S1: Comparative descriptions of available *in vitro* sensitivity assays for *Plasmodium falciparum***

(n.d.): no data.
