# Supplementary material for: A Novel Flow Cytometric Hemozoin Detection Assay for Real-Time Sensitivity Testing of Plasmodium falciparum
Source: PLoS One. 2013 Apr 24;8(4):e61606. doi: 10.1371/journal.pone.0061606 (PMC3634823; doi:10.1371/journal.pone.0061606)
Supplement: Table S2 — Antimalarial activities of several antimalarial drugs determined by different in vitro sensitivity assays against P. falciparum 3D7 strain. (DOCX) [file pone.0061606.s002.docx]

**Table S2: Antimalarial activities of several antimalarial drugs determined by different *in vitro* sensitivity assays against *P. falciparum* 3D7 strain**

|  | **[3]-Hypoxanthine incorporation** | **WHO schizont maturation test** | **SYBR green I**  **plate assay** | **HRP2 assay** |
| --- | --- | --- | --- | --- |
| **Quinine** | 63 nM [1] | 35 nM [1] | 6.9 nM [13] | 5.9 nM [13] |
|  | 102.3 nM [2] |  |  |  |
|  | 47.1 nM [3] |  |  |  |
|  | 59.9 nM [4] |  |  |  |
| **Mefloquine** | 42.6 nM [5] | 12.1 nM [11] | 9.4 nM [13] | 8.4 nM [13] |
|  | 6.1 nM [2] |  | 9.5 nM [14] |  |
|  | 18.2 nM [3] |  | 40.7 nM [12] |  |
|  | 34.5 nM [4] |  |  |  |
| **Chloroquine** | 15.7 nM [5] | 14.79 nM [7] | 8.1 nM [13] | 7.5 nM [13] |
|  | 6 nM [6] |  | 22.2 nM [15] | 9.7 nM [7] |
|  | 9.7 nM [2] |  | 16 nM [16] |  |
|  | 11.3 nM [3] |  | 11.54 nM [7] |  |
|  | 18.7 nM[4] |  |  |  |
|  | 9.6 nM [7] |  |  |  |
|  | 29.6 nM [8] |  |  |  |
| **Amodiaquine** | 20.3 nM [9] | 7.8 nM [1] |  |  |
| **Artemisinin** | 22 nM [5] |  | 9 nM [14] |  |
|  | 10.1 nM [10] |  |  |  |
| **Dihydroartemisinin** | 5.3 nM [5] |  | 3.78 nM [15] | 2.3 nM [17] |
|  | 4.2 nM [8] |  | 22.1 nM [12] |  |
| **Artesunate** | 1.1 nM [7] | 3.7 nM [7] | 1.9 nM [7] | 1.5 nM (48h) [7] |
|  | 9.4 nM [9] | 5.4 nM [12] | 3.6 nM [15] | 2.5 nM (72h) [7] |
|  | 0.4 nM [3] |  |  | 2.4 nM [17] |
|  | 0.9 nM [4] |  |  |  |
| **Atovaquone** | 1.3 nM [5] | 0.11 nM [7] | 0.06 nM [7] | 0.8 nM [7] |
|  | 1.1 nM [9] |  |  |  |
|  | 0.06 nM [7] |  |  |  |
| **Pyrimethamine** | 78.4 nM [2] | 32.2 nM (48h) [7] | 7.2 nM [14] | 26.6 nM (48 h) [7] |
|  | 5 nM [6] | 26.3 nM (72h) [7] | > 100 nM (48h) [7] | 53.9 nM (72h) [7] |
|  | 19.3 nM (48h) [7] |  | 23.9 nM (72h) [7] |  |
|  | 19.4 nM (72h) [7] |  |  |  |

[1] [Chong CR](http://www.ncbi.nlm.nih.gov/pubmed?term=Chong%20CR%5BAuthor%5D&cauthor=true&cauthor_uid=14609745), [Sullivan DJ Jr](http://www.ncbi.nlm.nih.gov/pubmed?term=Sullivan%20DJ%20Jr%5BAuthor%5D&cauthor=true&cauthor_uid=14609745) (2003) Inhibition of heme crystal growth by antimalarials and other compounds: implications for drug discovery. Biochem Pharmacol 66:2201-12.

[2] Vivas L, Rattray L, Stewart LB, Robinson BL, Fugmann B, et al. (2007) Antimalarial efficacy and drug interactions of the novel semi-synthetic endoperoxide artemisone in vitro and in vivo. J Antimicrob Chemother 59:658-65.

[3] Aunpad R, Somsri S, Na-Bangchang K, Udomsangpetch R, Mungthin M, et al. (2009) The effect of mimicking febrile temperature and drug stress on malarial development. Ann Clin Microbiol Antimicrob 8:19.

[4] Lim P, Wongsrichanalai C, Chim P, Khim N, Kim S, et al. (2010) Decreased in vitro susceptibility of Plasmodium falciparum isolates to artesunate, mefloquine, chloroquine, and quinine in Cambodia from 2001 to 2007. Antimicrob Agents Chemother 54:2135-42.

[5] Duraisingh MT, Roper, C, Walliker D, and Warhurst DC (2000) Increased sensitivity to the antimalarials mefloquine and artemisinin is conferred by mutations in the pfmdr1 gene of *Plasmodium falciparum*. Molecular Microbiology 36: 955-961.

[6] Reynolds JM, El Bissati K, Brandenburg J, Günzl A, Mamoun CB (2007) Antimalarial activity of the anticancer and proteasome inhibitor bortezomib and its analog ZL3B. BMC Clin Pharmacol 7:13.

[7] Wein S, Maynadier M, Tran Van Ba C, Cerdan R, Peyrottes S, et al. (2010) Reliability of antimalarial sensitivity tests depends on drug mechanisms of action. J Clin Microbiol 48: 1651–1660.

[8] Wong RP, Salman S, Ilett KF, Siba PM, Mueller I, et al. (2011) Desbutyl-lumefantrine is a metabolite of lumefantrine with potent in vitro antimalarial activity that may influence artemether-lumefantrine treatment outcome. Antimicrob Agents Chemother 55:1194-8.

[9] Johnson JD, Dennull RA, Gerena L, Lopez-Sanchez M, Roncal NE, et al. (2007) Assessment and continued validation of the malaria SYBR green I-based fluorescence assay for use in malaria drug screening. Antimicrob Agents Chemother 51:1926-33.

[10] Baniecki ML, Wirth DF, Clardy J (2007). High-throughput *Plasmodium falciparum* growth assay for malaria drug discovery. Antimicrob Agents Chemother 51, 716.

[11] Wisedpanichkij R, Chaijaroenkul W, Sangsuwan P, Tantisawat J, Boonprasert K (2009) In vitro antimalarial interactions between mefloquine and cytochrome P450 inhibitors Acta Trop 112:12-5.

[12] Wang Z, Parker D, Meng H, Wu L, Li J, et al. (2012) In vitro sensitivity of *Plasmodium falciparum* from China-Myanmar border area to major ACT drugs and polymorphisms in potential target genes. PLoS One 7:e30927.

[13] Bacon DJ, Latour C, Lucas C, Colina O, Ringwald P, et al. (2007) Comparison of a SYBR green I-based assay with a histidine-rich protein II enzyme-linked immunosorbent assay for in vitro antimalarial drug efficacy testing and application to clinical isolates. Antimicrob Agents Chemother 51:1172-8.

[14] Plouffe D, Brinker A, McNamara C, Henson K, Kato N (2008) In silico activity profiling reveals the mechanism of action of antimalarials discovered in a high-throughput screen. Proc Natl Acad Sci USA 05:9059-9064.

[15] He Z, Chen L, You J, Qin L, Chen X (2010) In vitro interactions between antiretroviral protease inhibitors and artemisinin endoperoxides against *Plasmodium falciparum*. Int J Antimicrob Agents 35:191-193.

[16] Ramalhete C, Lopes D, Mulhovo S, Molnár J, Rosário VE, et al. (2010) New antimalarials with a triterpenic scaffold from Momordica balsamina. Bioorg Med Chem 18:5254-60.

[17] Held J, [Soomro SA](http://www.ncbi.nlm.nih.gov/pubmed?term=Soomro%20SA%5BAuthor%5D&cauthor=true&cauthor_uid=21392942), Kremsner PG, Jansen FH, Mordmüller B (2011) In vitro activity of new artemisinin derivatives against *Plasmodium falciparum* clinical isolates from Gabon. Int J Antimicrob Agents 37:485-488.
