# Supplementary material for: A Novel Flow Cytometric Hemozoin Detection Assay for Real-Time Sensitivity Testing of Plasmodium falciparum
Source: PLoS One. 2013 Apr 24;8(4):e61606. doi: 10.1371/journal.pone.0061606 (PMC3634823; doi:10.1371/journal.pone.0061606)
Supplement: Figure S1 — Cyflow® flow cytometer and optical bench layout. Images in the top row show the components (A) and the optical bench layout (B) of the Cyflow® flow cytometer. The 488 nm laser light is vertically polarized. A horizontally polarized filter is placed in front of a second side scatter detector to allow detection of depolarized light (depol SSC). Images C, D and E show a Cyflow® in the laboratory and being easily packed for transport at the Centre de Recherches Médicales de Lambaréné – CERMEL, Lambaréné, Gabon. (The subject of the photograph has given written informed consent, as outlined in the PLOS consent form, to publication of the photograph). (DOCX) [file pone.0061606.s003.docx]

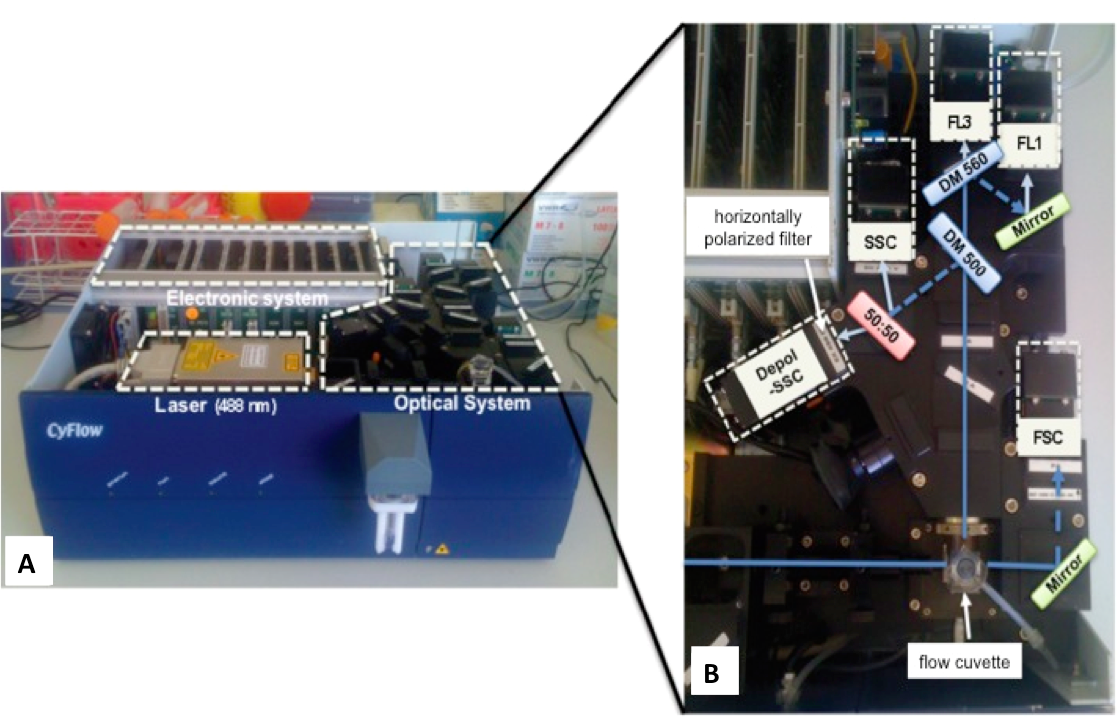


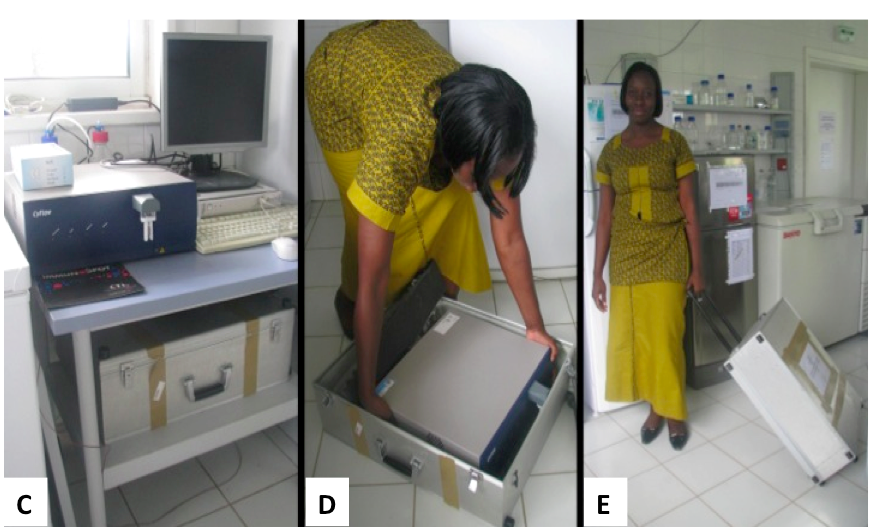


**Figure S1: Cyflow® flow cytometer and optical bench layout**

Images in the top row show the components (A) and the optical bench layout (B) of the Cyflow® flow cytometer. The 488 nm laser light is vertically polarized. A horizontally polarized filter is placed in front of a second side scatter detector to allow detection of depolarized light (depol SSC). Images C, D and E show a Cyflow® in the laboratory and being easily packed for transport at the Centre de Recherches Médicales de Lambaréné – CERMEL, Lambaréné, Gabon.

(The subject of the photograph has given written informed consent, as outlined in the PLOS consent form, to publication of the photograph).
